# A Systematic Review of Wi-Fi and Machine Learning Integration with Topic Modeling Techniques

**DOI:** 10.3390/s22134925

**Published:** 2022-06-29

**Authors:** Daniele Atzeni, Davide Bacciu, Daniele Mazzei, Giuseppe Prencipe

**Affiliations:** Department of Computer Science, University of Pisa, Largo B. Pontecorvo 3, 56127 Pisa, Italy; davide.bacciu@unipi.it (D.B.); daniele.mazzei@unipi.it (D.M.); giuseppe.prencipe@unipi.it (G.P.)

**Keywords:** machine learning, Wi-Fi, BERTopic, topic modeling, artificial intelligence

## Abstract

Wireless networks have drastically influenced our lifestyle, changing our workplaces and society. Among the variety of wireless technology, Wi-Fi surely plays a leading role, especially in local area networks. The spread of mobiles and tablets, and more recently, the advent of Internet of Things, have resulted in a multitude of Wi-Fi-enabled devices continuously sending data to the Internet and between each other. At the same time, Machine Learning has proven to be one of the most effective and versatile tools for the analysis of fast streaming data. This systematic review aims at studying the interaction between these technologies and how it has developed throughout their lifetimes. We used Scopus, Web of Science, and IEEE Xplore databases to retrieve paper abstracts and leveraged a topic modeling technique, namely, BERTopic, to analyze the resulting document corpus. After these steps, we inspected the obtained clusters and computed statistics to characterize and interpret the topics they refer to. Our results include both the applications of Wi-Fi sensing and the variety of Machine Learning algorithms used to tackle them. We also report how the Wi-Fi advances have affected sensing applications and the choice of the most suitable Machine Learning models.

## 1. Introduction

The advent of the first wireless connections radically changed our ever-growing and ever-evolving society. Nowadays, mobiles, tablets, and laptops, with their ability to easily have Internet access, represent indispensable tools for a large number of people. Wireless Local Area Networks (WLANs) have become ubiquitous, and they are now essential to people’s professional and personal lifestyles.

Among the variety of wireless communication technologies, Wi-Fi has played a fundamental role since its birth, becoming the dominant model of wireless Internet access today. In 2019, more than three-billion Wi-Fi-enabled devices have been shipped [1], and it is estimated that Wi-Fi’s share of Internet traffic will grow by 51% in 2022 [2]. Nowadays, Wi-Fi is the most appropriate choice for WLANs, due to the more reliable and cost-effective wireless connections with the higher data rate it provides in indoor environments.

Some recent innovations have even increased the interest and potential of this technology. The advent of Internet of Things (IoT), with its multitude of physical interconnected objects exchanging data coming from their sensors, brought Wi-Fi applications to a new level. With the development of IoT infrastructures, Wi-Fi can regulate the communication of the majority of objects in our houses and cities, our healthcare devices (Internet of Medical Things), and industrial machines (Industrial IoT). Regarding the last one, the formalization of the Fourth Industrial Revolution, or Industry 4.0 (I4.0), could lead in future years to the spread of fully automatized dark factories, in which Wi-Fi will play a key role [3].

Finally, the constant evolution of Wi-Fi standards and technologies has enabled its durability, allowing it to last more than 30 years. Among the innovation brought by Wi-Fi, honorable mentions go to high-speed optical communications, multiple-input multiple-output (MIMO), and orthogonal frequency-division multiplexing (OFDM) transmission technologies, which allowed faster and more reliable communications [4]. Other important aspects that Wi-Fi has to face are security and privacy. These issues led to the development of novel encryption standards, such as Wi-Fi Protected Access and anonymization techniques.

The growth in the number of devices using Wi-Fi to connect to wireless networks has led to an increase in the pervasiveness of Wi-Fi signals. The abundance of these signals has resulted in the development of techniques that exploit connectivity data for various tasks. These techniques created a new research field called Wi-Fi sensing [5] and exploit the information contained within each message, e.g., the signal intensity. Studying the signal intensity of each message can give information about the position of the emitting device, and the analysis of the variation of the signal allows us, for example, to understand the behavior or the motion of a user. The enormous spread of Wi-Fi-enabled devices, however, makes these analyses challenging both for the amount and the variability of the available data. To overcome these problems, another technological breakthrough of the last couple of decades can become helpful: Machine Learning (ML).

Machine Learning no longer needs an introduction. It has proven itself useful in almost every aspect related to technology, and the global interest in it suggests its future potential. ML applications have reached unthinkable performances in, for example, computer vision [6], Natural Language Processing (NLP) [7], and competitive games [8]. ML algorithms are a flexible and efficient way to analyze an arbitrary—quite often large—amount of data coming from a big variety of sources. Its ability to generalize and to adapt to every situation, as long as the data used during the training of ML algorithms are well-representative for the given task, makes these techniques helpful in a wide range of applications.

For these reasons, it should not be surprising that Wi-Fi connections and their signal intensity represent another application for Machine Learning algorithms. In recent years, applications involving both Wi-Fi and Machine Learning have multiplied, giving rise to a variety of independent research fields. Although this specialization has led to great improvements in individual fields, it is increasingly difficult to navigate through their multitude. The goal of this systematic review is to give a broader perspective on the applications of Machine Learning on Wi-Fi connection data. By doing so, it is possible to help both new researchers who are interested in finding their way in such a wide space and experts in the field to search for possible solutions in fields similar to their own.

In particular, in this work we aim at answering the following research questions:

1. Which tasks and applications relative to Wi-Fi signals have been tackled with Machine Learning techniques?
2. What are the most widely used Machine Learning methods applied to Wi-Fi data?
3. How did this field of research develop with respect to the evolution of Wi-Fi technology?

Given the breadth of the analysis we want to perform, in this work we exploit an NLP technique, topic modeling [9], to obtain a preliminary classification of the papers presented in the literature. This technique has proven itself useful in clustering and extracting meaningful insight from a corpus of documents [10,11]. The groups obtained after this preliminary step will ease our analysis and allow us to examine a wider variety of articles.

The rest of the paper is organized as follows: In Section 2, we present an overview of similar works presented in the literature; Section 3 offers an introduction on the Wi-Fi technology and ML techniques; in Section 4, we describe the methodology and algorithms used in this work; Section 5 reports the results of the topic modeling phase; in Section 6, we answer the proposed research questions; finally, in Section 7, we summarize our work and results.

## 2. Related Works

The vastness of topics covered in this work and the possible different points of view imply the presence of various reviews. However, these primarily focus on specific applications or technologies.

In the context of applications, one of the most popular uses of Machine Learning for Wi-Fi data is indoor positioning, and a plethora of reviews and surveys on this topic are present in the literature. These works give different points of view and perspectives. Some of them are more general [12,13,14] and differ from each other mainly for the review’s procedure. Others analyze in more detail the data source, differentiating between channel state information [15] and received signal strength [16]. The literature also offers surveys on Machine Learning techniques that leverage Wi-Fi data to face human fall detection [17], human activity recognition [18], smart homes [19], motion detection [20], and human mobility [21]. Despite being useful for understanding specific tasks, these reviews fail to provide a more general overview of the usefulness of ML in the Wi-Fi context.

There also exist surveys and reviews which shift the focus from applications to particular settings or technologies. For example, ref. [22] aims at analyzing the use of Machine Learning in UAV-based communications. In [23], the authors study the use of Machine Learning to improve Wi-Fi performance, by finding the best configuration of WLANs parameters to optimize network performances.

In the literature, there are also works with more general views. In [5], the authors categorize articles using various dimensions, i.e., signal processing, algorithm, application, and performance, but focusing only on the channel state information. In [24], the evolution of Wi-Fi technology is described, driven by the authors’ personal lens. They also report some possible Wi-Fi applications without considering the methods used to fulfill them.

Our systematic review differs from the others in the literature, to the best of our knowledge, because of its wider scope, not being limited to any particular application or technology. In this work we aim at understanding how Machine Learning techniques have been used in previous works in relation to Wi-Fi, i.e., using data coming from characteristics of Wi-Fi connections as input. We also differ from the majority of the existing reviews in the literature in this context, as far as we know, by the methodology we used. We will adopt an NLP to to cluster a variety of articles into fewer and more easily interpretable groups. This technique has be proven to obtain good results in other works, such as [10,11], and we think that it could be helpful for analyzing other popular and rapidly growing research fields.

## 3. Preliminaries

In this section, we provide some preliminary notions that will be helpful in the rest of the paper. We firstly describe Wi-Fi technology and some of its changes over the years. We also outline some of the difficulties that handling Wi-Fi data implies. Then we introduce some general notions about Machine Learning and artificial intelligence, and some of the most popular models and algorithms.

### 3.1. The Wi-Fi Technology

Wi-Fi technology is now part of our daily lives: mobiles, laptops, and smart TVs are just some examples of devices that make use of it, and the advent of the Internet of Things can only lengthen this list. Its ease of use and the continuous drop in the price of chipsets for Wi-Fi contributed strongly to this expansion. The devices use Wi-Fi to communicate via radio signals over the airwaves with an access point (AP), a piece of networking hardware connected to a wired network or a cellular network using the tethering technique. The AP essentially converts data conveyed through the Internet into radio waves and broadcasts them into the surrounding environment.

**Figure 1:**
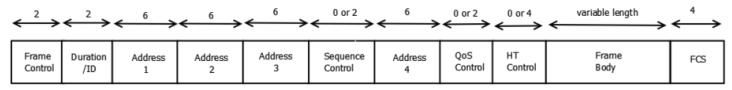
A standard 802.11 frame. Figure obtained from https://en.wikipedia.org/wiki/802.11_Frame_Types, accessed on 20 April 2022.

The communication standard is a subset of the IEEE 802 protocol family. It provides several distinct radio frequency ranges, which vary between 2.4 and 60 GHz [25,26,27] and defines the organization of data packets, also known as frames. The frames are composed of several fields, shown in Figure 1, that facilitate the management of sharing the same access point between various devices. The most important fields regarding the communication are the MAC addresses, which identify both the source and the destination of each data packet. Whenever a transmission is received, the receiver looks at the destination MAC address and determines whether the transmission should be ignored or not.

The ease of access to the wireless network is one of the biggest advantages of Wi-Fi, but it also represents a serious issue when it comes to security and privacy. A possible attacker could attack multiple devices just by being within the range of the Wi-Fi network. Moreover, the communication between a user device and an AP could lead to serious privacy leakage. The first of the two problems is constantly evolving and has led to the creation of various encryption standards over the years. This process has resulted in the creation of Wired Equivalent Privacy (WEP), Wi-Fi Protected Access (WPA), and finally WPA2, which is the current encryption method adopted by Wi-Fi networks. Regarding the second problem, one of the major issues is represented by probe requests (PRs), which will be analyzed in detail in the next section.

#### Probe Requests

The IEEE 802.11 defines the set of protocols and standards for implementing wireless local area networks (i.e., WLANs), specifically the “media access control” layer and the physical layer [26]. In the media access control layer, for short MAC, endpoints communicate with each other using frames. Frames are used both as a means to communicate and to manage a WLAN: management frames do everything from authentication to discovering access points. The way mobile devices discover new stations is using what is called a probe request. A PR, as specified in the IEEE 802.11 standard, is a request of information from a station to another station, and the answer is called a *probe answer*. How station discovery is achieved is very simple: a device sends a probe request in a broadcast on the radio, and all the access points which received it answer it. A station can also show itself using a beacon frame. However, the probe approach is less energy-consuming and is preferred: instead of always listening for a beacon frame on the radio, the device keeps the radio on just for a few milliseconds, just in time to receive the probe response it needs. As for the probe request frequency, it has been shown that bursts frequently happen, even when a device is locked with the Wi-Fi option turned on [28]. Being broadcasted on the radio, everyone can read these frames, which usually contain very important information about the device that sent them. Each frame is composed of a header, a payload, and a frame check sequence. The address used in the header to identify the destination is called the MAC address, which needs to be unique in the same network. This need for the address to be unique has brought to the creation of the IEEE Registration Authority, which assigns and manages the “organizationally unique identifiers” (OUI): to each organization it is given a unique 24-bit OUI, which is then used to create an extended unique identifiers (EUI). The EUI are used for applications that require fixed-size globally unique identifiers, such as network interfaces, but as the IEEE also states: “The IEEE Registration Authority makes a concerted effort to avoid duplicate assignments but does not guarantee that duplicate assignments have not occurred. Global uniqueness also depends on the proper use of assignments and the absence of faults that might result in duplication” [29]. Saying that a MAC Address is globally unique to a device is incorrect, but it helps track devices across multiple networks.

### 3.2. Wi-Fi as a Data Source

The ubiquity of Wi-Fi-enabled devices and APs represents a continuous source of data. Every time a connection between two devices is established, the radio wave characteristics of the data exchange can provide a variety of useful features. Unfortunately, this feature extraction does not come for free: for their nature, radio wave signals present a huge variety of problems.

First of all, the enormous diffusion of Wi-Fi connections makes it difficult to isolate the radio waves coming from a target device, permeating every possible application location with background noise and adding variability to the data.

Moreover, being a radio wave, the Wi-Fi signal depends on a variety of factors: the frequency band, radio power output, receiver sensitivity, antenna gain, antenna type, and modulation technique. For example, changing between an omnidirectional antenna and a semi-parabolic antenna can change the range of an AP from 100 m to more than 30 km. A change in any of these factors translates into data variability, increasing the difficulty of possible applications. Additionally, the environment plays a fundamental role in signal propagation. APs and devices are immersed in a dynamic environment, where the signal can reflect, refract, or diffract due to buildings, trees, cars, or moving people. Despite providing useful and exploitable environmental information, these waves characteristics must be taken into account and thwarted whenever we want to use Wi-Fi signals.

Finally, radio waves suffer from interference. The signal coming from Wi-Fi devices can collide with the ones coming from non-Wi-Fi devices which share the 2.4 GHz band, such as microwave ovens, security cameras, and Bluetooth devices. The congestion of certain channels can become a problem in high-density areas, such as large apartment complexes or office buildings with many Wi-Fi access points, and affect the quality of the Wi-Fi data we want to exploit. A signal can also interfere with itself in the phenomenon called the multi-path effect. Among the causes of this phenomenon, there are atmospheric ducting and reflection from water bodies or solid objects, such as buildings and mountains. This effect results in signals reaching the receiving antenna by more than one path, causing both constructive and destructive interference and phase shifting. The multi-path effect causes jitter and ghosting, for example, in analog television and GPS receivers, and can also lower the goodness of the incoming data.

Despite these problems, these radio waves contain much useful information. In the next sections, we describe two of the most used features of Wi-Fi data.

#### 3.2.1. Received Signal Strength

The received signal strength indicator (*RSSI*) is a measurement of the power present in a received radio signal. In particular, in an IEEE 802.11 system, RSSI is the relative received signal strength in a wireless environment, in arbitrary units. RSSI is an indication of the power level being received by the receiving radio after the antenna and possible cable loss. Therefore, the greater the RSSI value, the stronger the signal. It is possible to estimate the physical distance between the transmitter and the receiver via a path loss model, as described in [30]. The relationship between the distance *d* and the RSSI is given by:

(1) $$RSSI=RSSI_{0}-10nlog_{10}\left(\frac{d}{l_{0}}\right)+X_{\sigma }$$

where $RSSI_{0}$ is the signal power at a reference distance $l_{0}$, *n* is the path loss exponent which depends on the physical environment, and $X_{\sigma }$ is normally distributed random noise with 0 mean and σ standard deviation. Using this relationship, it is possible to obtain a formula for the distance given the RSSI as:

(2) $$d=10^{(RSSI_{0}-RSSI)/10n}.$$

Machine Learning approaches that deal with Wi-Fi signals typically use RSSI at different APs to associate each device with a fingerprint. These fingerprints are then used as input for an ML model that solves a given task.

#### 3.2.2. Channel State Information

The adoption of the latest wireless telecommunication innovations has allowed Wi-Fi() data to go beyond the simple RSSI. In particular, the combination of multiple-input multiple-output antennas and orthogonal frequency division multiplexing lead to the adoption of the channel state information as the data source. In its simplest form, the CSI is a complex matrix with one row for each transmitting antenna and one column for each receiving antenna. When the OFDM also plays a role in the telecommunication setup, the CSI matrix becomes a tensor with an additional dimension representing the various subfrequencies into which the channel is divided. The goal of the CSI is to capture information about the surrounding environment and the effects it produces, i.e., multipath propagation and fading. To estimate its entries, periodical streams of known sequences are transmitted from the source to the destination. By comparing the received and the input signals, it is possible to estimate a matrix for each subcarrier that allows representing the received signal vector $y_{i}$ as

(3) $$y_{i}=H_{i}x_{i}+n_{i},$$

where $x_{i}$ is the input signal vector and $n_{i}$ is a noise vector, usually sampled from a normal distribution [5].

Given its objectives and characteristics, it should not surprise the reader that CSI could be very useful when we want to capture changes in an environment. The wireless signals’ sensitivity to people reflects variations in CSI that have shown themselves really helpful in indoor localization [31], gesture recognition [32], and user authentication [33].

### 3.3. Overview of Machine Learning

Machine Learning (ML) [34] refers to a variety of statistical models that, given a dataset, are able to automatically tune their parameters to reflect patterns and structures hidden in the data. These learning techniques are usually divided into three categories, based on the kinds of tasks they address.

Supervised learning [35] comes into play when the dataset is composed of two parts, the input data and the target. The goal of supervised models is to find a function that maps the input data into target values that minimize a user-defined *loss* function. The loss function depends on the type of the target variable. If the target variable can assume a finite number of alternative values, i.e., we are tackling a *classification* task, the most popular loss function is cross entropy [36]. Instead, if we are predicting one or more real-value outputs, we may use mean-squared error or mean-squared logarithmic error as the loss function. On the contrary, unsupervised learning [37] models are used to find patterns within unlabeled data, i.e., data where there is no prior information about the expected model target. Relevant instances of unsupervised learning are clustering techniques and generative models. In the former, the algorithm seeks groups, or clusters, of data, in order to categorize them and ease their analysis. The latter are statistical models used to understand the factors that characterize and generate the data. Finally, reinforcement learning [38] refers to situations in which we want an agent to perform a sequence of actions (a policy) in order to maximize a reward function. The focus of reinforcement learning is on finding a balance between exploration of new and unknown situations and the exploitation of agent current knowledge.

Note that this partition is not strict. In fact, for example, there are approaches that combine ideas from both unsupervised and supervised learning. For instance, autoencoders [39] and variational autoencoders [40] are particular models with supervised training whose unsupervised objective is the generation or modification of the input data. They achieve their goal by having the same input and target data (or some slightly modified version of them) and minimizing a particular loss called reconstruction loss. Besides this rough but necessary partition, in recent decades a wide variety of models have been created that are able to handle all sorts of input data (e.g., time series, images, and graphs). In the following we describe in more detail some of the most popular Machine Learning and artificial intelligence models.

#### 3.3.1. The Neural Network and Its Descendants

**Figure 2:**
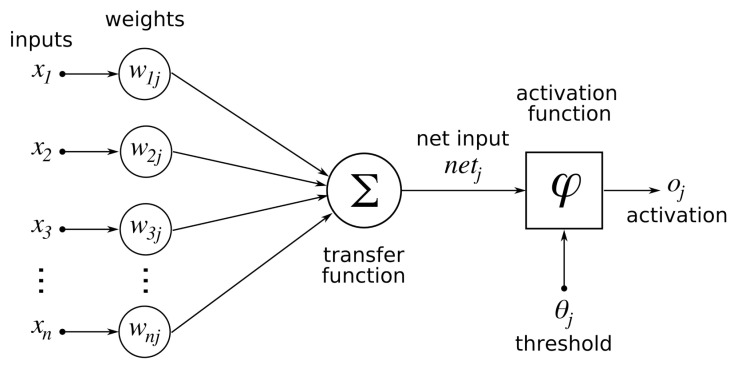
Computational schema of a neuron in a neural network. The input values are aggregated (usually by a weighted sum) and then a non-linear activation function is applied. Figure obtained at https://commons.wikimedia.org/wiki/File:ArtificialNeuronModel_english.png, accessed on 20 April 2022.

Neural Networks (NNs) are the most popular, studied, and developed Machine Learning models. They were introduced in 1934 by McCulloch [41], who took inspiration from humans’ biological neurons. They consist of layers of neurons, or computation units, connected together by weights. Each neuron aggregates the values coming from nodes of the previous layer based on their connection weights. Then, it uses a non-linear *activation* function to compute the output value and propagates it to the next layer. Figure 2 represents the computational schema of a neuron. Despite initial inactivity due to the computational constraints of the period, they gained popularity with the introduction of the backpropagation algorithm [42], which allows one to train NNs, i.e., find the ideal values of the weights, quickly and efficiently.

Basic differentiation between neural networks is based on the network topology. The simplest structure is the Multi-Layer Perceptron (MLP), in which each neuron of a layer is connected to every neuron in the previous and the next layer. If feedback connections are present, the model is called Recurrent Neural Network (RNN) [43], and it is typically used to handle sequential data. RNNs have also been extended to more articulated forms of neural units to avoid typical problems, e.g., the difficulty in learning long-term dependencies. The most popular development of RNNs, in this sense, are Long-Short Term Memory (LSTM) [44] and the Gated Recurrent Unit (GRU) [45]. Another kind of model that is widely used on sequential data (although being originally devised for multi-sets) is the transformer [46]: it adopts a particular mechanism to focus on significant parts of the sequence, called self-attention. Among the most popular neural networks variations, we can cite the Convolutional Neural Networks (CNNs) [6], which are designed to handle images and exploit a mathematical operation, the convolution, to reduce the number of learnable parameters of the network while architecturally enforcing spatial invariance properties.

#### 3.3.2. K-Nearest Neighbors

K-Nearest Neighbor (K-NN) [47] is a supervised learning algorithm that classifies unseen input data according to the class of the k closest seen data. This simple algorithm only requires a definition of distance in the input data space and the number of neighbors to be considered. The classification can be done in a variety of ways, including the most frequent class among the neighbors or via a weighted contribution of the neighbors, in which the weight of each neighbor is inversely proportional to the distance.

#### 3.3.3. Support Vector Machine

The Support Vector Machine (SVM) [48] is a linear model that creates the *ideal* hyperplane to separate the classes. The term “ideal” here means that the hyperplane selected is the one that maximizes the distances between the hyperplane and the closest elements of each class. SVMs also adopts a technique that allows one to find a solution also for non-linearly separable data. Moreover, by using the so-called kernel trick, SVMs can look for an hyperplane in a higher-dimensional feature space in an efficient way. By using this trick, the input data are mapped in a new space in which, ideally, the input data are linearly separable.

#### 3.3.4. Decision Tree and Random Forest

The decision tree (DT) [49] is an easily interpretable model that produces a tree-structured sequence of decision nodes and leaves. Each decision node divides the dataset into subsets according to the value of one of the features. The value and the feature of each decision node are learnt during the training phase. The leaves are instead responsible for predicting the target value for an input data. It is also possible to prune the tree after the training process, to avoid too complex and overfitted models.

The random forest (RF) [50] is the evolution of decision trees. As the name suggests, a random forest is composed of a multitude of decision trees. The target value is then predicted by aggregating the contribution of each decision tree. The aggregation of a lot of decision trees makes the model more robust and less prone to overfitting.

## 4. Review’s Methodology

**Figure 3:**
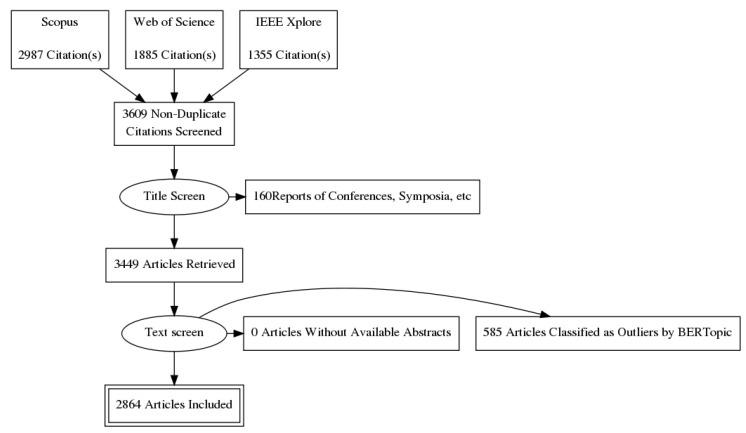
Workflow of this systematic review, following the one described in the PRISMA statement.

This review follows the methodology described in the PRISMA statement [51]. The complete diagram is shown in Figure 3. In the following, we describe and explain in more detail the various steps of the review.

### 4.1. Data Retrieving and Screening

The analyzed articles have been downloaded on the 14th of March 2022 from three different sources, the Scopus [52] and the Web of Science [53] databases, and the IEEE Xplore digital library [54]. All of these sources are quite popular and frequently updated; they offer APIs to easily query them and have been used for similar works in the past [12]. The Scopus and IEEE Xplore search engines allow searching for strings inside titles, abstracts, and keywords; the Web of Sciences search engine allows only searching inside the abstract. In each case, the string searched was the following:

(“wifi” OR “wi-fi”) AND (“machine learn*” OR “deep learn*” OR “artificial int*” OR “neural net*” OR “svm” OR “decision tree” OR “knn”)

where the * indicates the presence of zero or more alphanumerical characters.

The query returned, respectively, 2987, 1885, and 1355 articles for Scopus, Web of Sciences, and IEEE Xplore. The results of the queries were then merged, and duplicates articles removed, thereby obtaining 3609 articles. Additionally, Scopus API returned results that contain all the papers presented during a conference or symposium. The titles of these results are the names of the event they summarize. Thus, we filtered the articles whose titles include one of the following words:

- conference;
- workshop;
- symposium;
- meeting;
- forum;

The final dataset was composed of 3449 papers.

#### Dataset Exploration

**Figure 4:**
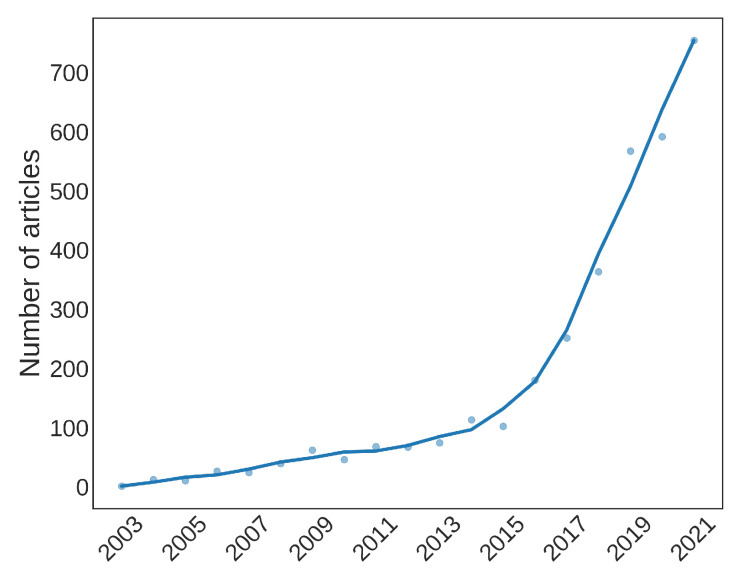
Number of articles retrieved for each year.

After the data retrieving, we can begin our analysis with some considerations about metadata. Figure 4 shows the article count for the last 20 years. The exponential trend is very clear, and it reflects well both the increasing interest and spread of Wi-Fi-enabled devices, and the exploitation of the huge amount of data they provide.

With respect to the academic interest, the total number of citations of the 3449 papers is more than 27,000; each paper was cited 7.97 times on average. The most cited papers are reported in Table 1. It is interesting to note how these articles differ in both the topics covered and the points of view analyzed. This fact highlights the breadth of possible application of Machine Learning and the pervasiveness of Wi-Fi in our lives.

Table 2 reports the types of papers retrieved from the databases. More than half of the results (56%) are composed of conference papers. Articles and proceeding papers combined form 42% of the results.

It is also interesting to notice that the same process repeated on articles related to Wi-Fi only returned a little more than 34,000 results. Thus, the co-occurrence of both Wi-Fi and ML terms was responsible for less than the 12% of the works about Wi-Fi.

### 4.2. Topic Modeling

The amount of available text data has produced an incredible spread of Natural Language Processing (NLP) inside the Machine Learning world. In order to ease the analysis of the huge number of text documents, the birth and development of topic modeling took place. Usually, topic modeling techniques are unsupervised Machine Learning algorithms to automatically detect phrase patterns and group together sets of documents well-represented by the same set of co-occurring words and expressions.

A variety of techniques have been developed over the years. Among the most popular methods, one needs to mention Latent Semantic Analysis (LSA) [62] and Latent Dirichlet Allocation (LDA) [63]. The former refers to a sequence of statistical analysis of terms frequency, where each document is treated as a bag of words. The second one is a generative model that assumes words’ distribution over a document as a finite mixture of an underlying set of topic distributions.

In our work, we use BERTopic [64], a recent pipeline which exploits word embeddings and real-valued vector clustering algorithms. In the next sections, we describe in more detail the text preprocessing and the various steps of this method.

#### 4.2.1. Data Preprocessing

Before moving to the actual topic modeling phase, we also performed some preprocessing procedures on the texts of the abstracts. To do this, we applied a standard data preparation pipeline, common to most of the NLP tasks, which consisted of the following steps:

- Tokenization, i.e., splitting the text into tokens, usually into single words.
- Stop words’ removal, including both English stop words (e.g., “the”, “is”, “which”) and ad hoc non-discriminative words: “Wi-Fi”, “method”, “paper”, etc.
- Lemmatization, that is, the process of reducing a term to its root, e.g., “are” and “am” become “be”, and “better” becomes “good”.
- N-gram extraction, i.e., sequences of n words from a sample of the text that satisfy statistical constraints. In this work we use unigrams, bi-grams, and tri-grams.

Although these steps are not strictly necessary with the used topic modeling algorithm, we noticed a big improvement in the results with preprocessed abstracts. In particular, removing ad hoc stop words allows the algorithm to disregard common and general words and better discriminate topics. At this point, the text corpus can be used as input for the topic modeling algorithms.

#### 4.2.2. The BERTopic Algorithm

BERTopic [64] is a recent framework for topic modeling composed of three steps which leverages word embeddings, feature reduction, and classical clustering algorithms.

The first step is the mapping of words and documents into a real-value vector. As the name suggests, the original version of this frameworks adopts the popular BERT model [65] to obtain meaningful representations of the documents, but any of the modern deep learning models for NLP can be used. In our work, we stuck with the original proposal and adopt BERT.

Similarly, the second step can be performed with any feature reduction algorithm, such as principal component analysis (PCA) [66] or TSNE [67]. The goal of this intermediate step is to decrease the number of features of the embeddings and avoid the curse of dimensionality, thereby easing the work of the clustering phase. In the first proposal of BERTopic, the authors used UMAP [68], an algorithm that exploits differential geometry and algebraic topology concepts to preserve the global structure of the vectors in the lower dimensional space; we used the same approach here.

Finally, a clustering algorithm is used to group the documents into topics. Again, the choice of the clustering technique is arbitrary: in our work this part was carried out by Hierarchical DBSCAN (HDBSCAN) [69]. This algorithm combines the advantages of hierarchical clustering methods with DBSCAN: instead of taking a cut level as a hyperparameter, as in standard DBSCAN, HDBSCAN allows varying density clusters by looking at the most stable groups during a hierarchical split process. Like DBSCAN, it also allows one to automatically recognize noise data, and it does not require prior knowledge of the ideal number of clusters.

In conclusion, BERTopic takes advantage of the latest and advanced deep learning models for NLP to cluster documents into topics. Despite not being specifically designed for this task, it allows great flexibility in each of its steps, provides easily understandable results, and does not require any prior knowledge of the number of topics.

#### 4.2.3. Results Interpretation

After obtaining the clusters, we need to analyze the results. To do that, the first step is to identify the most representative words for each topic. This task is achieved by modifying the Term Frequency-Inverse Document Frequency (TF-IDF), a classic score to find the relevance of a word in a collection of documents. The standard formula for TF-IDF of the term *i* in document *j* is

$${\left(TF-IDF\right)}_{i,j}=\frac{n_{i,j}}{|d_{j}|}\times \log\frac{\left|D\right|}{1+|\{d\in D:i\in d\}|}$$

where $n_{i,j}$ is the number of occurrences of term *i* in document *j*, $|d_{j}|$ is the length of document *j*, and *D* is the set of documents (one is added to the denominator of the logarithm argument just to avoid division by zero). The first of the two factors is just the frequency of a given term in a document, and the second one measures the importance of the term in the collection of documents, i.e., whether it is common or rare in the overall corpus.

In order to identify the importance of a word within a topic, we considered the documents forming a topic as a single document. In this case, the first term of the TF-IDF is the frequency of a given term in a topic, and the second one detects the importance of the term across all the topics. This metric is called class-based TF-IDF (c-TF-IDF).

Embeddings are also useful for other analyses. For example, if we want to find the most appropriate topic for a given term, we can simply compute the word embedding and compare it with topic embeddings. The closer the two embeddings are, the more similar is the topic to the provided term.

The last analysis we performed on our results focused on identifying the most representative documents for each topic: to do that, we could use the λ values returned by HDBSCAN. For each point in the dataset, i.e., each document embedding, the λ value was bigger for the points that persisted the most during the hierarchical splitting process; hence, it represented the strength of its cluster membership.

### 4.3. Reproducibility

Regarding the computer tools, Python was used for the analyses and figure creation. Specifically, regarding libraries NumPy, Pandas, and xlrd were used to load and explore the data downloaded from the various sources; SpaCy was adopted for the text preprocessing phase; Bertopic (https://github.com/MaartenGr/BERTopic) and the libraries with which it works (PyTorch, scikit-learn, and UMAP) were employed for extracting the topics; and Matplotlib, Seaborn, and WordCloud were used to visualize the results and produce the figures. The raw and preprocessed data, and the Python notebooks, are available in this GitHub repository: https://github.com/daniele-atzeni/A-Systematic-Review-of-Wi-Fi-and-Machine-Learning-Integration-with-Topic-Modeling-Techniques.

## 5. Topic Modeling Results

After running the topic modeling phase, we obtained nine clusters. The number of papers for each cluster is reported in Table 3, along with articles detected as noise elements, identified as members of Topic −1.

**Figure 5:**
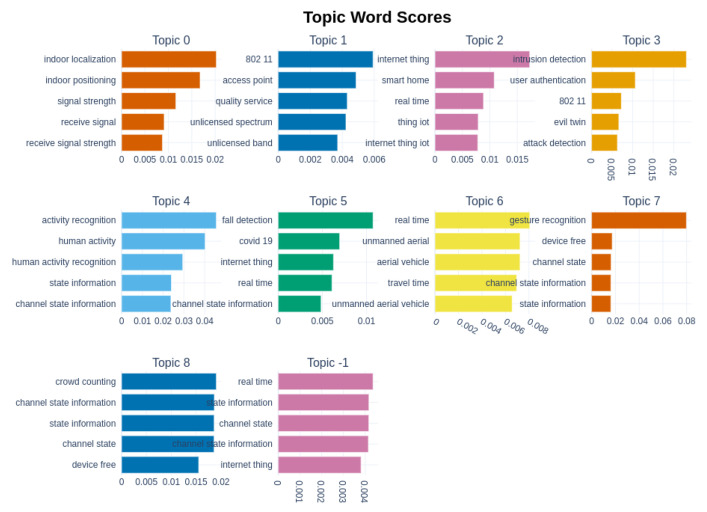
List of the topics with the most relevant terms (ordered by c-TF-IDF).

The most relevant words, considering their c-TF-IDF, are shown in Figure 5. The largest topic by far, containing one-third of the documents in the corpus, refers to Indoor Localization. Given the size of this topic, we tried another round of topic modeling to identify possible subtopics, but the discriminant words between these subtopics were only related to the type of data and the ML model used. We will better analyze these factors in Section 6.

Figure 5 also allows us to appreciate the clarity of the obtained results. In fact, the majority of the topics are easily understandable by looking at their most representative terms. The only result that is difficult to interpret is Topic 1. To better understand it, we ran a manual investigation of the papers, showing the presence of applications of Machine Learning for improving wireless connections. Among the most recent papers, there are [70], in which a Machine Learning solution for solving the line-of-sight discovery problem in indoor mmWave Wi-Fi networks is proposed. Another example is [71], where the authors compare various Machine Learning algorithms to detect symbols in orthogonal frequency-division multiplexing transmissions. The three most representative documents for this topic are [72,73,74]. These three works face, from different perspectives, the problem of optimizing the quality of service of a wireless network with the help of Machine Learning models for resource allocation. The cited papers and a further in-depth analysis suggest that this topic is related to the use of ML techniques to improve or understand the use of Wi-Fi connections and wireless infrastructures.

The terms of Figure 5 and the previous considerations about the results were used to assign each topic to the following representative names:

- Topic 0: Indoor Localization
- Topic 1: ML for Improving Wireless Networks’ Performances
- Topic 2: IoT and Smart Houses
- Topic 3: Privacy and Intrusion detection
- Topic 4: Human Activity Recognition
- Topic 5: Human Condition Monitoring
- Topic 6: Wi-Fi and ML for improving UAVs networks
- Topic 7: Gesture Recognition
- Topic 8: Crowd Monitoring and People Counting

**Figure 6:**
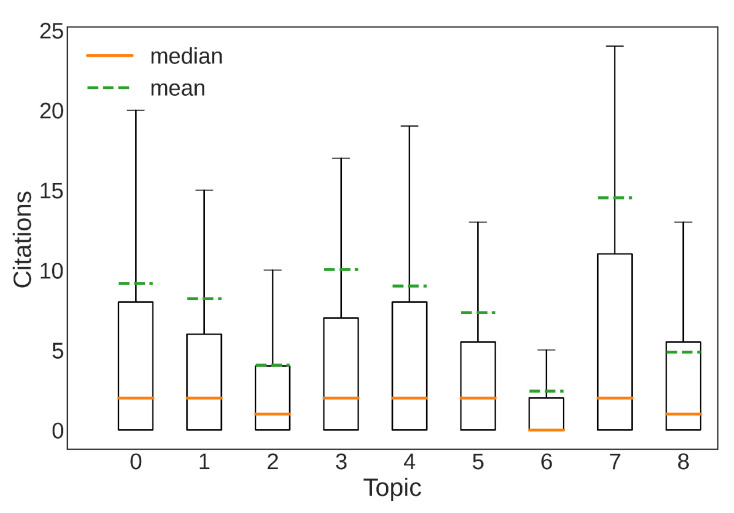
Boxplots obtained by the number of citations of the documents, grouped by topic.

To conclude this section, we analyze the article citations. Figure 6 shows boxplots representing the distributions of the numbers of citations of the articles in each topic. This image gives a clear idea of the importance of human-related applications, such as gesture recognition and human activity recognition. Surprisingly, topics related to IoT and robotics seem to attract less interest. It seems that the combination of Wi-Fi connections data and Machine Learning has not yet been appreciated in these contexts, despite the continuous growth of research fields about IoT and Industry 4.0.

## 6. Answers to the Research Questions

In this section, we try to answer the research questions introduced in Section 1.

### 6.1. RQ 1

*Which tasks and applications relative to Wi-Fi signals have been tackled with Machine Learning techniques?*

To answer this question, we refer to Section 5. In fact, among the clusters described in that section, we can identify the tasks that have been faced with Machine Learning. We can divide the articles’ clusters obtained by BERTopic into two main categories. The first one, including topics 0, 4, 5, 7, and 8, contains articles that aim at studying mostly human-related contexts. Topics 1, 2, and 6 focus more on the type of infrastructure in which these applications have been deployed. Topic 3 is positioned in the middle of these two categories and will be better analyzed later in this section.

**Figure 7:**
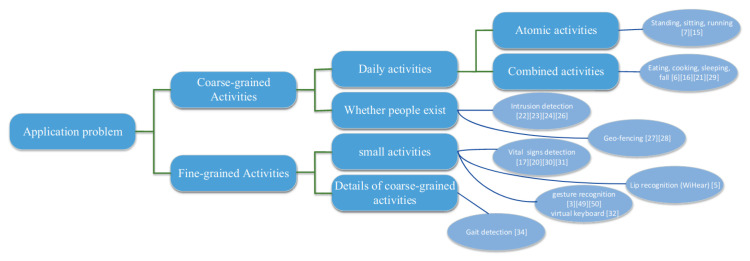
Human-related activity classification by Ma et al. [75].

With respect to the human-related studies, an interesting work on these applications is the survey by Ma et al. [75], where the authors divided these activities into coarse-grained activities and fine-grained ones (Figure 7). The first term refers to macro-level activities, such as actions (e.g., running, sitting, or cooking) or presence detection. The second ones are more specific and require more controlled environments, such as monitoring vital signs or sleep quality analysis by looking at a patient’s breath or heartbeat. Other than Indoor Localization, the first group contains three other topics identified by BERTopic, i.e., Human Activity Recognition, Gesture Recognition, and Crowd Monitoring and People Counting. Among this group and the overall topics, indoor positioning and localization is the most popular research field that adopts both Wi-Fi and ML. The performance drop of GPS-based techniques in indoor environments justifies the interest related to this field. A variety of techniques have been developed throughout the years. Typically, ML models are usually fed with real-valued vectors constructed from the measurement of devices’ Wi-Fi signals, called fingerprints. A nice survey on this technique and its application in indoor localization is [76]. These techniques can also be divided into active and passive ones. Active positioning refers to the ability to locate users having a device that is actively searching for nearby APs. On the contrary, passive positioning techniques have the ability to understand the location by looking at the changes in the propagation of the signal affected by the presence of a user. A comprehensive survey on the topic is given by [77].

Human Activity Recognition and Gesture Recognition also have attracted a lot of interest, based on their topic sizes and numbers of citations, respectively. Interestingly, from the most relevant terms of these topics shown in Figure 5, we can note the importance of CSI for these tasks. The finer granularity of CSI data with respect to RSSI has allowed a variety of methodologies and algorithms to be applied to device-free sensing applications, which is well summarized in [78].

On the contrary, Crowd Monitoring and People Counting seem less relevant than other similar applications. Despite a recent boost in their numbers of publications (two-thirds of the article in this topic happened in the last three years), the statistics on the citations suggest that this field has not reached yet its full potential. In fact, while GPS-based data have been widely used for analyzing outdoor crowd behaviors [79], the same cannot be said about the use of Wi-Fi data for its indoor counterpart. These kinds of studies could give, for example, interesting insights about social behaviors, e.g., social dynamics in schools or workplaces, by being less invasive than other technologies, such as video-based ones.

The second group described by [75] is well represented in Topic 5. This topic groups together the studies in which Machine Learning algorithms have been used to control human health parameters, such as heart rate and body temperature, and to detect falls, which is among the major threats for elderly people [17]. Despite Wi-Fi connections providing meaningful information in this scenario and allowing one to obtain encouraging results both on their own [80,81] and in combination with other kinds of sensors [82], this topic seems to be yet under-explored.

Regarding the category of papers focused on infrastructures, we have already analyzed Topic 1 in Section 5. Topics 2 and 6 have the worst results in terms of citations, as highlighted by Figure 6. A possible explanation for this phenomenon is the technical challenges that these topics present. In fact, using Machine Learning in IoT scenarios (Topic 2) is quite challenging because of the low computational power and memory capacity of IoT devices [83]. Topic 6 is related to the adoption of UAVs and drones for communication purposes, and shows many technical difficulties regarding interference, resource management, and channel modeling [22].

Finally, Topic 3, Privacy and Intrusion Detection, is positioned in the middle of the two categories. By manually looking at the latest most cited and most representative papers, we noticed that articles in this topic could be further divided into two distinct groups. The first and seemingly bigger group is related to intrusion detection in wireless networks. In fact, ML algorithms have, for example, proven useful for identifying both spoofing attacks [84] and evil twins [85]. Reference [86] provides a comprehensive survey about this specific group. The second group focuses on authentication of users and users’ actions. This aspect is clearly related to the previous one, since identifying a user and its behavior implies intrusion detection. However, this broader task brings up privacy issues, which in the past led to solutions such as MAC address randomization. Examples of this second group are in [87,88], in which the authors identified users’ actions through Machine Learning techniques to analyze user-AP interactions and IoT devices (smart refrigerators, TVs, etc.), respectively.

### 6.2. RQ 2

*What are the most widely used Machine Learning methods applied to Wi-Fi data?*

To answer this question, and to understand whether there is any correspondence between methods and tasks, we computed the frequencies of specific Machine Learning models both inside the complete dataset and the topics. We search the papers for the following keywords and/or their acronyms:

- Neural Networks, even if this term refers to a superset that includes the following models;
- Convolutional Neural Networks;
- Recurrent Neural Networks, for which we also used the terms Long-Short Term Memory and Gated Recurrent Unit;
- Transformers;
- Support Vector Machines;
- K-Nearest Neighbors;
- Random forests and decision trees.

Table 4 reports the occurrences of these words within the topics and the complete dataset. As we can see, Neural Networks are the most used models by far, appearing more than three times than SVM and KNN, and more than four times more than random forests and decision trees. Among the different neural models, the most used are CNNs and RNNs. Transformers have found less applications, possibly due to their recent in formalization and diffusion.

**Figure 8:**
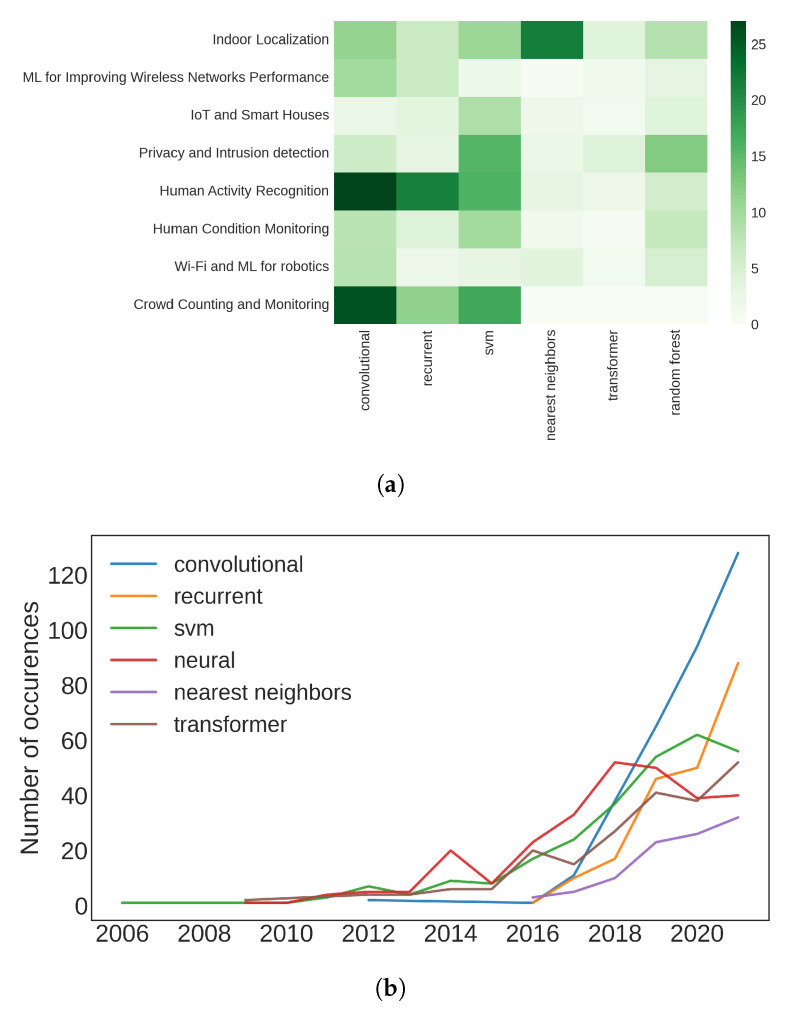
Graphs representing the Machine Learning models counts grouped by topics (**a**) and years (**b**). The term “Neural Network” is not present in order to have more comparable values. (**a**) Heatmap of the occurrences of ML models. (**b**) Word hits of the different ML algorithms over the years.

Figure 8a shows the frequency of each model in each topic with respect to topics size. We removed neural networks from the image to have clearer comparisons between models. The heatmap brings out the importance of K-Nearest Neighbors in Indoor Localization. In fact, one of the most popular techniques to locate devices in indoor environments is the application of KNN using device fingerprints and a dataset of offline measured reference points [89,90]. We can also notice the wide use of CNNs and RNNs for Human Activity and Gesture Recognition. This fact should not surprise, since they are two of the most popular architectures for Neural Networks. RNNs are also specifically designed for time series analysis and perfectly match tasks that try to understand evolving phenomena such as Gesture Recognition. On the contrary, the use of CNNs is more mysterious. It is not clear whether their popularity is related to a lack of knowledge in these interdisciplinary scenarios [91], or to their capability of extracting relevant features from multiple data streams, e.g., subcarrier in MIMO communications.

Another interesting insight is given by Figure 8b, which again witnesses to the Neural Network’s popularity. We can in fact notice how classical ML algorithms, i.e., K-NN, SVM, and random forest, have been rapidly overcome by the advent of more advanced Machine Learning models and NNs.

### 6.3. RQ 3

*How did this field of research develop with respect to the evolution of Wi-Fi technology?*

**Figure 9:**
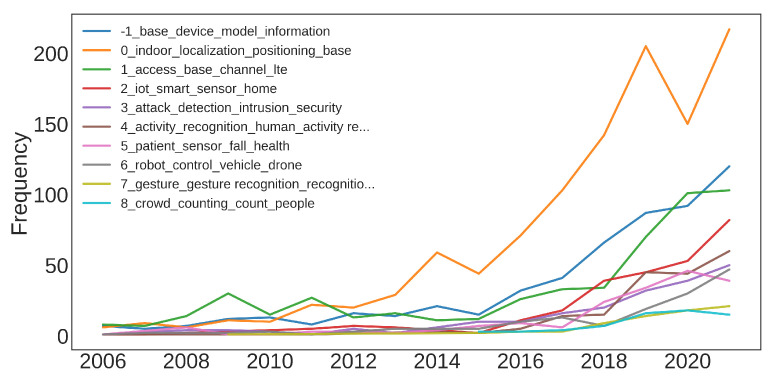
Number of articles published over the years for every topic.

The first way we can analyze to answer this question is the number of articles published in each year for every topic found in Section 5 (Figure 9). From this figure we can appreciate better the focus of the researchers in the last decade on indoor localization. It is also interesting to note the rapid growth in recent years of the topic “ML for Improving Wireless Networks’ Performances”. This growth in interest is possibly due to the recent advancements in cellular networks and machine-to-machine communications and network congestion, caused by the spreading of wireless devices.

**Figure 10:**
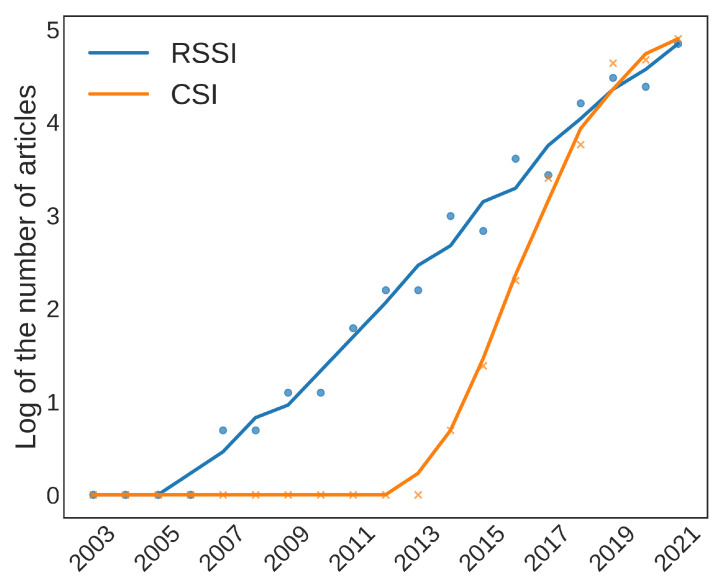
Count of the number of occurrences of CSI and RSS in the logarithmic scale.

Another interesting source of information is connected to how ML applications reacted to the advent of CSI. Figure 10 shows the logarithms of the numbers of occurrences of the terms “RSS” and “CSI”. The logarithmic scale allows us to compare the growth of these two terms. As we can see, the late introduction of the CSI did not stop it from reaching and overcoming the number of uses of RSSI. The CSI spread also correlates with the rise in the number of applications that use CNNs, as shown in Figure 8b. In fact, CNNs seem to be able to extract relevant features from the multiple CSI sub-carrier channels, as described in [92].

Finally, we wanted to understand how ML applications that use Wi-Fi data have been influenced by the introduction of randomized MAC addresses in probe requests. Unfortunately, this topic seems to be still little explored. In fact, only 53 articles contain the words “probe”, “mac addr”, or “randomized”, with a total 597 of citations (for an average of 11.3 citations for each paper). Moreover, several of them focuses on studying the real effects of MAC randomization [93,94] or device de-anonymization [95,96]. There are in the literature some works that leverage PRs, e.g., in crowd detection [97] and behavior [98] or device classification [99], but we believe that there is still room for improvement.

## 7. Conclusions

In this paper, we presented a systematic review of the applications of Machine Learning models for Wi-Fi connectivity data analysis. The aim of the work was to understand the possible applications of Wi-Fi data that can take advantage of the flexibility of Machine Learning and its ability to analyze a huge amount of data. We also wanted to understand how the rapid evolution of these research fields affected their interactions.

In order to analyze a bigger number of articles, we adopted a recent topic modeling technique, i.e., BERTopic, that leverage word embeddings and clustering techniques to extract meaningful topics from text data. The obtained topics clearly show the variety of fields that have been influenced by the combination of Wi-Fi data and Machine Learning. The field that has exploited this type of data by far is indoor positioning, but Wi-Fi’s ubiquity has facilitated progress also in human activity and gesture recognition, privacy, and intrusion detection. The topics highlight also the technologies involved, which vary from IoT and smart houses to UAVs and wireless networks. We also highlighted possible under-explored fields of research, such as indoor crowd monitoring and people counting, and pioneering and challenging studies, such as including UAVs in wireless communication infrastructures.

In our results, we also reported a comparison between the usage of different ML techniques. We highlighted the growth in popularity of neural network architectures with respect to classical ML algorithms. We also compared different models and algorithms grouping them both by topic and year: we showed that the K-Nearest Neighbors algorithm is still widely adopted for indoor localization in combination with the fingerprint technique; and Convolutional Neural Networks and Recurrent Neural Networks have taken over in human activity and gesture recognition.

Finally, we analyzed the role of Wi-Fi innovations, i.e., CSI and randomized MAC addresses, showing the continuous growth in the numbers of applications of RSSI and CSI. In particular, CSI, combined with CNNs and RNNs, has seen a steep increase in the number of publications that cited it, and has assumed a dominant role as data source. We also noted that probe requests have not attracted much interest compared to the former, possibly because of randomized MAC addresses and privacy-preserving techniques.

## Figures and Tables

**Table 1:** List of the most cited papers.

First Author	Year	Reference	Citations
Andrews J.G.	2012	[55]	950
Wang X.	2017	[56]	583
Ferris B.	2007	[57]	415
Pan S.J.	2008	[58]	367
Dimatteo S.	2011	[59]	261
Kolias C.	201	[60]	218
Zhao M.	2018	[61]	216

**Table 2:** Numbers of different paper types and their percentages.

Paper Type	Number of Papers	Perc
Conference Paper	1943	56%
Article	1173	34%
Proceeding Paper	269	8%
Chapter	34	1%
Review	30	1%
Others	9	-

**Table 3:** Topics counts obtained with BERTopic and their relative proportions with respect to the whole dataset.

Topic	Count	Perc
0	1136	33%
1	537	16%
2	280	8%
3	218	6%
4	200	6%
5	191	6%
6	160	5%
7	72	2%
8	70	2%
−1	585	17%

**Table 4:** Count of the number of occurrences of various ML models for each topic.

Topic	Size	NN	CNN	RNN	Transf	SVM	KNN	RF
0	835	196	29	46	12	80	206	74
1	529	161	54	33	10	11	3	16
2	354	160	89	61	8	50	8	15
3	270	47	6	10	3	23	6	11
4	211	44	12	7	14	32	5	27
5	194	51	15	8	2	20	2	13
6	156	35	13	2	2	6	6	7
7	138	102	57	7	33	7	7	2
8	80	30	25	7	6	16	5	4
−1	682	187	59	46	14	49	39	48
Tot	3449	1013	359	227	104	294	287	217

## Data Availability

The code and data used to produced the results and images are available in the following GitHub repository: https://github.com/daniele-atzeni/A-Systematic-Review-of-Wi-Fi-and-Machine-Learning-Integration-with-Topic-Modeling-Techniques. The data have been downloaded with the previously described queries from https://www.scopus.com/search/form.uri?display=basic#basic (Scopus), https://www.webofscience.com/wos/woscc/basic-search (Web of Science), and https://ieeexplore.ieee.org/search/advanced (IEEE Xplore).
